# Investigation of the Relationship between Electronic Structures and Bioactivities of Polypyridyl Ru(II) Complexes

**DOI:** 10.3390/molecules28135035

**Published:** 2023-06-27

**Authors:** Zhiying Hou, Yang Lu, Bin Zhang, A. F. M. Motiur Rahman, Yufen Zhao, Ning Xi, Ning Wang, Jinhui Wang

**Affiliations:** 1 Institute of Drug Discovery Technology (IDDT), Ningbo University, Ningbo 315211, China; 2 Li Dak Sum Yip Yio Chin Kenneth Li Marine Biopharmaceutical Research Center, Department of Marine Pharmacy, College of Food and Pharmaceutical Sciences, Ningbo University, Ningbo 315800, China; 3 Department of Pharmaceutical Chemistry, College of Pharmacy, King Saud University, Riyadh 11451, Saudi Arabia

**Keywords:** ruthenium complex, phenanthroline, electronic structure, cytotoxicity, bioimaging, off-on

## Abstract

Ruthenium (Ru)-based organometallic drugs have gained attention as chemotherapeutic and bioimaging agents due to their fewer side effects and excellent physical optical properties. Tuning the electronic structures of Ru complexes has been proven to increase the cytotoxicity of cancer cells and the luminescent efficiency of the analytical probes. However, the relationship between electronic structures and bioactivities is still unclear due to the potential enhancement of both electron donor and acceptor properties. Thus, we investigated the relationship between the electronic structures of Ru(II) complexes and cytotoxicity by optimizing the electron-withdrawing (complex **1**), electron-neutral (complex **2**), and electron-donating (complex **3**) ligands through DFT calculations, bioactivities tests, and docking studies. Our results indicated that it was not sufficient to consider only either the effect of electron-withdrawing or electron-donating effects on biological activities instead of the total electronic effects. Furthermore, these complexes with electron-donating substituents (complex **3**) featured unique “off-on” luminescent emission phenomena caused by the various “HOMO-LUMO” distributions when they interacted with DNA, while complex with electron-withdrawing substituent showed an “always-on” signature. These findings offer valuable insight into the development of bifunctional chemotherapeutic agents along with bioimaging ability.

## 1. Introduction

Due to its antiproliferative activities, cisplatin served as the first metal-based anticancer drug and it is still being applied in clinical treatment nowadays. Unfortunately, the resistance and side effects of cisplatin, such as ototoxicity, gastrointestinal toxicity, nephrotoxicity, and hepatotoxicity, constrained its efficacy in the clinical application [1]. Later, the modification of platinum (Pt)-based drugs was established to reduce the side toxicities, for example, Carboplatin, Oxaliplatin, Nedaplatin, and others [2,3,4]. Nevertheless, all of these Pt-based chemotherapeutics show unsatisfactory substantial advantages over cisplatin. Therefore, many researchers began looking into alternative metal-based drugs such as iridium (Ir) complexes, osmium (O_S_) complexes, and rhenium (Re) complexes, finding out that ruthenium (Ru) complexes could be great potential chemotherapy agents with low systemic toxicities and high bioactivities according to in vivo/in vitro studies [5,6,7,8,9,10]. The interaction of Ru-based drugs with DNA and proteins is primarily responsible for their mechanism of action, which is highly dependent on the configuration and conformation of these complexes to modulate the interaction efficiency, lipophilic, solubility, and other properties. Among the Ru-based complexes, NKP-1019/IT-139 and NAMI-A were the most promising anticancer drug candidates which were currently undergoing clinical trials [11,12,13]. In addition to the conventional Ru-based chemotherapy agents, photodynamic therapy (PDT) was emerging as a novel treatment due to the fascinating inter-system crossing under light excitement between the singlet metal-ligand charge transfer (^1^MLCT) state and triplet metal-ligand charge transfer ^3^MLCT state, which could transfer energy to ^3^O_2_ and produce reactive oxygen species (ROS, such as ^1^O_2_, O_2_^−^, •OH…), resulting in toxicity and leading to cell death [14,15]. Another, a polypyridyl complex TLD-1433, was evaluated in a phase II clinical trial against bladder cancer using PDT as a photosensitizer [16,17]. Additionally, photothermal therapy (PTT) and photoactivated chemotherapy (PACT) were both attractive therapeutics that were brought about by the conversion of light to thermal energy via nonradiative relaxation pathways to restrain the tumors, and by the photodissociation of ligands to toxicity agents (Ru aqua complex, the free ligand, or both), respectively [18,19,20,21]. In addition, Ru(II)-based photosensitizers (Ps) traditionally played an essential role in the detection of biomolecules in vitro and in vivo at the cellular/molecular level due to their unique optoelectronic properties such as long-lived triplet state [22,23,24]. Based on those, abundant Ru-based molecules, including arene complexes, heteronuclear complexes, and polypyridyl complexes, have been established and studied for their biological functions.

In light of these, it becomes crucial to comprehend the physical properties of Ru-based metallodrugs, particularly with regard to their electronic structures. Yet, there was still some ambiguity regarding how these polypyridyl Ru(II) complexes’ bioactivities related to their electronic structure. For instance, Rilak’s team reported that the chelate ligands with electron donors would accelerate the hydrolysis rate to form the corresponding adducts with biomacromolecules, producing potent antitumor effects [25]. Gasser’s group claimed that the Ru(II) polypyridyl core with the electron-donating group preferred negatively charged semiquinonate ligands and benefitted the cytotoxicity of Ru(II) complexes toward multiple targets [26]. Very recently, we have also demonstrated that tuning the electron-donating ability of 1,10-phenanthroline by amino substituents could enhance the interaction with certain protein residues and DNA to increase cytotoxicity [27]. However, in stark contrast to the description above, the electron-withdrawing group also contributed significantly to the promotion of antitumor activity. For instance, Liu’s group studied thoroughly the electron-donating and electron-withdrawing groups in half-sandwich Ru(II) N^N (aryl-BIAN) complexes, both displaying outstanding antiproliferative potency while the electron-withdrawing substituents (-F) decorated Ru-complexes elicited the best cytotoxicity through lysosome-mediated apoptosis [28]. Ajibade’s group proposed that the electron-withdrawing group in these complexes was highly crucial for their receptor interactions via noncovalent bonding (i.e., hydrogen bonding) [29]. The ambiguous impact of electron donors and acceptors in Ru(II) complexes on anticancer activity fascinated researchers, prompting them to explore the exact mechanism of metallodrugs. Moreover, the “off-on” characteristic for the promising bioimaging probes was well correlated with the modifications made to the electronic structures as a result of the reaction or interaction with the target molecules. However, the construction of these probes typically includes difficult synthetic processes, such as: (i) precise leading receptors to the target organelle or tissue allowing reliable guidance to aiming objectives to be tested; (ii) smart sensing linkers facilitating “off-on” luminescence response towards targets; (iii) effective luminescence core owing forceful penetration and high quantum yield to avoid the disturbing tissue fluorescence and to increase the efficiency of the probe [30]. Understanding the effects of electron-donating and electron-withdrawing groups is important because the entire discussion up to this point heavily relies on the electronic properties of the Ru(II) complexes.

To investigate the relationship between the electronic structures and bioactivities of polypyridyl Ru(II) complexes, we designed and synthesized Ru (II) complexes with electron-withdrawing group (-Cl), electron-neutral group (-H), and electron-donating group (-OCH_3_) optimized 1,10-phenanthroline ligands constructed Ru(II) complexes, namely, [Ru(tpy)(Cl-phen)Cl](PF_6_) (**1**), [Ru(tpy)(phen)Cl](PF_6_) (**2**), and [Ru(tpy)(MeO-phen)Cl](PF_6_) (**3**). The electronic properties of the substituents were predicted by electronic surface potential (ESP), frontier molecular orbital (FMO) energy levels, and bond dissociation energies (BDE) according to density functional theory (DFT) calculations, which were also identified by spectroscopic studies. In addition, interaction with *L*-histidine, DNA-binding results, and MTT tests of these Ru(II) complexes straightforwardly revealed that it should not only consider the electron-donating or -withdrawing effect to optimize the interaction effect with clear target motifs such as *L*-histidine and N_7_ in guanosine but also the total electronic effects in the oriented pocket. Intriguingly, we found the abnormal “off-on” phenomenon only existed in the situation of electron-donating substituents modified Ru(II) complexes, which was caused by the distribution change of HOMO and LUMO during DNA-binding studies. Whereas, the electron-withdrawing groups optimized Ru(II) complexes featured an “always-on” emission. Consequently, systematically modulating the electronic groups in Ru(II)-based drugs paves the way toward understanding the further development of such metallodrugs in cancer treatments and analytical detection.

## 2. Results and Discussion

### 2.1. Density Functional Theory (DFT) Calculations

**Figure 1:**
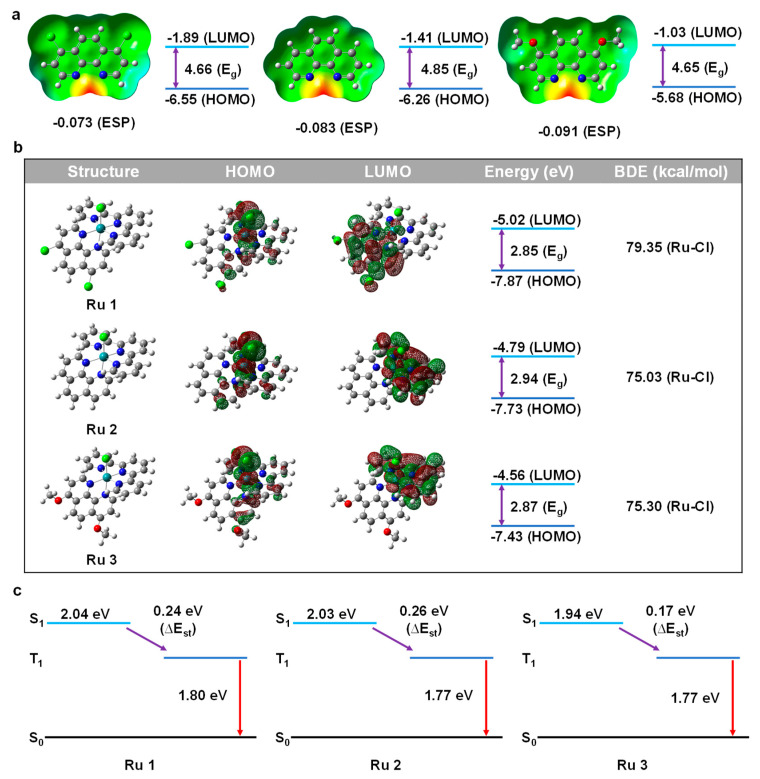
(**a**) ESP (red part represents the most nucleophilic part) and FMO calculation of phenanthroline derivatives. (**b**) HOMO and LUMO distribution and energy calculation of Ru complexes. (**c**) TD−DFT calculation of Ru complexes.

We conducted computational research using DFT methods on various substituents of phenanthroline ligands since DFT calculation is an effective and precise method to reveal the physical properties of drugs design (p-Cl-phen, phen, p-MeO-phen). Electrostatic surface potential (ESP), is often known as a molecule’s capacity for coordination, or its capacity of the electron-donating ability [31]. Comparing the ESP absolute value could allow us to objectively assess the nucleophilicity and better understand the impact of substituents. As illustrated in Figure 1a, the absolute ESP value of these ligands was increased from −0.073 a.u. (for p-Cl-phen) to −0.083 a.u. (for phen) and −0.091 a.u. (for p-MeO-phen), respectively. The larger negative values mean stronger nucleophilicity, reflecting the electron-donating abilities of 1,10-phenanthroline ligands. Furthermore, the energy level of the highest occupied molecular orbitals (HOMOs) and the lowest occupied molecular orbitals (LUMOs) also suggested the electronic effect of various substituents. All the energy levels of these HOMOs and LUMOs became shallower along with the increasing electron-donating ability of the functional groups, in which the HOMOs were increasing from −6.55 eV to −6.26 eV and −5.68 eV for complex **1** to complex **3,** respectively. Meanwhile, the energy levels of LUMOs were raised from −1.89 eV to −1.41 eV and −1.03 eV for complex **1** to complex **3**, respectively. It is interesting to note that the energy gaps (E_g_) did not exactly match the electron-donating properties of these substituents, with complex **2** having the greatest E_g_ (4.85 eV). Further, we investigated the distribution of HOMO/LUMO, bond dissociation energy (BDE), and time-dependent density functional theory (TD-DFT) studies in order to learn more about the photophysical characteristics of complex **1** to complex **3** as shown in Figure 1b,c. It was clear that all the HOMOs of these complexes were mainly located on the chlorine (Cl), ruthenium (Ru), and the axial partial phenanthroline rings towards “Cl-Ru”. The LUMO of complex **2** and complex **3** were primarily centered on the terpyridine moieties, while the LUMO of complex **1** was mostly located on the 1,10-phenanthroline portion. What should be noticed is that terpyridine ring and phenanthroline ring are both ligands that could give electrons (i.e., all of them could anticipate in the HOMO distribution), so the HOMO distributions were the whole energetics of the entire system. However, for the ligand with an electron-withdrawing group (-Cl), it weakened the electron-donating ability of phenanthroline, which resulted in the difference in LUMO distributions. These various distributions of HOMO and LUMO discussed above were also significant phenomena that had a direct impact on the luminescence, which will be covered in more detail in the section on bioimaging. With pristine phenanthroline ligands, the energy level of HOMO/LUMO also followed the same rule, getting shallower as the nucleophilicity increased. Additionally, the *E*_g_ of complex **3** was 2.87 eV compared to complex **1** (2.85 eV) and complex **2** (2.94 eV), which was in line with the *E*_g_s of pristine ligands. What should be noticed is that the *E*_g_ values in Figure 1a only represent the energy gap of the pristine ligands, while the *E*_g_ values in Figure 1b state the total energy gap of the complexes. The comparison of these two different *E*_g_ values helps us to understand the total electronic effect better. The hydrolysis of Cl on Ru to form bonds with DNA and certain protein residues is one of the generally acknowledged antitumor actions of the Ru(II) complex, hence it was important to calculate the BDE of Ru-Cl. Although the BDEs were reduced with the increasing electron-donating ability of the phenanthroline ligands, the value of complex **2** is slightly less than the value of complex **3**, which may be due to the larger dipole moment (7.20 Debye for complex **2** and 7.09 Debye for complex **1**). One more thing, as we just mentioned, the PDT and PTT heavily rely on the triplet energy (*T*_1_). A tiny energy gap between singlet and triplet state (∆*E*_st_) would benefit the charge transfer from singlet (*S*_1_) to triplet (*T*_1_). Furthermore, it stands to reason that increasing the electron-donating ability of the ligands could reduce the ∆*E*_st_ and potentially enhance the PDT and PTT effects of these particular metal-based drugs [32].

### 2.2. Characterization, and Spectral Properties

**Figure 2:**
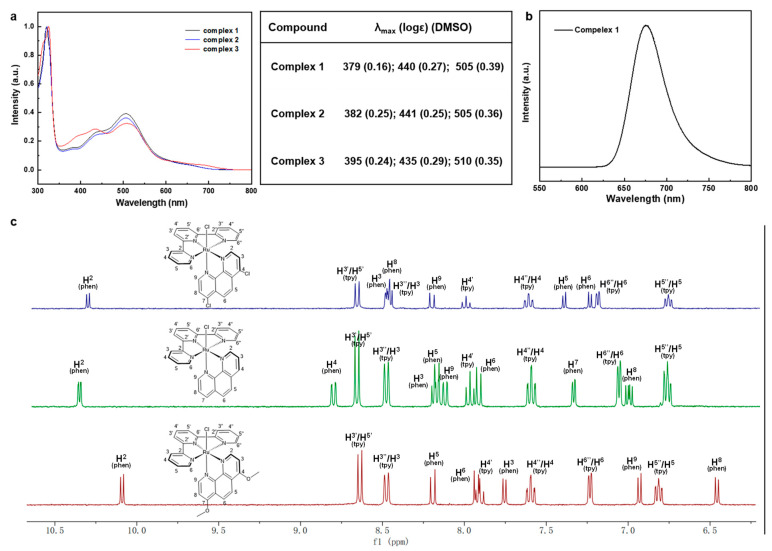
(**a**) UV absorption spectrum and data of Ru (II) complexes. (**b**) Emission spectrum of complex **1**. (**c**) ^1^H-NMR (aromatic part) of Ru (II) complexes.

Ultraviolet-visible (UV-Vis) absorption spectra were acquired in CH_3_CN and normalized for complexes **1**–**3** to compare the spectra differences as depicted in Figure 2a,b. Two types of absorption peaks were in the spectra, of which one was ligand to ligand charge transfer (LLCT) absorption, and another was metal to ligand charge transfer (MLCT) absorption. Strong absorptions in near-UV regions below 350 nm were attributable to traditional Ru(II) complexes ligand-cantered (LC) π–π* transitions, while broad absorptions regions at 379–395 nm, 435–441 nm, and 505–510 nm matched to Ru(dπ) → ligand (π*) MLCT for typical Ru(II) complexes. Simple observations showed that there were minor bathochromic shifts from complex **1** to complex **3**, which were entirely consistent with the predicted increased nucleophilicity of 1,10-phenanthroline derivatives. The red-shift values were consistent with the narrowing ∆*E*_g_ and ∆*E*_st_ discussed above, which benefit the electronic transition for complex **3**. Interestingly, only complex **1** displayed the apparent luminescent emission peak at 675 nm, which could be attributed to the well HOMO-LUMO distribution brought on by the electron-withdrawing effect of 4,7-dichloro-phenanthroline. It was challenging to evaluate the electronic effect solely by contrasting the electrochemical optical properties of these complexes, therefore we looked at the ^1^H NMR spectrum to investigate the proton chemical shift. The chemical shift of ^1^H NMR proved a reliable indicator of the compounds’ electronic characteristics, particularly for the electron densities on each proton and carbon. The chemical shift of these complexes was a roughly up-field shift along with the increasing nucleophilicity of the ligands according to the DFT calculations. Specifically, it is worth noting that the distance between H^2^ in phenanthroline (H^2^-phen) and chlorine (-Cl) was the smallest by only 2.795 Å. Thus, strong depletion of electrons occurred due to the large electronegativity of Cl, resulting in the least down-field shift. While the uttermost H^8^ in phenanthroline (H^8^-phen), which was brought about by the electron density accretion through para-directing activators (4,7-position’s EDGs) and outlying Cl, generated the greatest up-field shift. Therefore, the chemical shift of H^2^-phen and H^8^-phen could be compared to evaluate the electron density of these substituents. As shown in Figure 2c, there was no doubt that complex **3** owned the most up-field shift for both H^2^ (10.06 ppm) and H^8^ (6.95 ppm) compared with the other two complexes. It should be noted that the H^2^ was orientated towards chlorine (Ru-Cl), causing shielding to be impacted by the strong electron-withdrawing group, which led to the least down-field shift. Such a character could be an indicator to compare the electronic effect caused by the inductive and mesomeric effects of these substituents. Specifically, complex **1** with electron-donating group (-OCH_3_) would enrich the electron density in the pyridine ring, especially for the *para* position (H^2^), leading to the most up-shifted spectra. However, the chemical shift did not follow the rules as we expected in the situation for complex **1** and complex **2**. The chemical shifts of H^2^-phen were 10.27 ppm and 10.31 ppm for complex **1** and complex **2**, respectively; while the chemical shifts of H^8^-phen were not easy to figure out since the structure relationship was quite different from complex **3**. In addition, to evaluate the electron-donating ability of the ligands only, we simply compared the chemical shifts of the phenanthroline ring because the donor-acceptor systems were reversed. Further, we checked the COSY-spectrum of these complexes and assigned the peaks point by point as illustrated in Figures S4–S6. Intriguingly, the chemical shift of terpyridine ligands for complex **1** and complex **2** almost remained the same value except for H^6″^/H^6^ (tpy), but the chemical shifts of phenanthroline parts varied a lot. For example, we compared the six different types of peaks, and half of them are more up-shifted than others (H^3^, H^8^, and H^9^ are more up-shifted than complex **1**, while H^2^, H^5^, and H^6^ are up-shifted more than complex **2**). Combining the studies of DFT-calculations, UV-Vis absorption, and ^1^H NMR spectra, the electron-donating ability of these substituents basically followed the order (-OMe > -H > Cl).

### 2.3. Bioactivities

**Figure 3:**
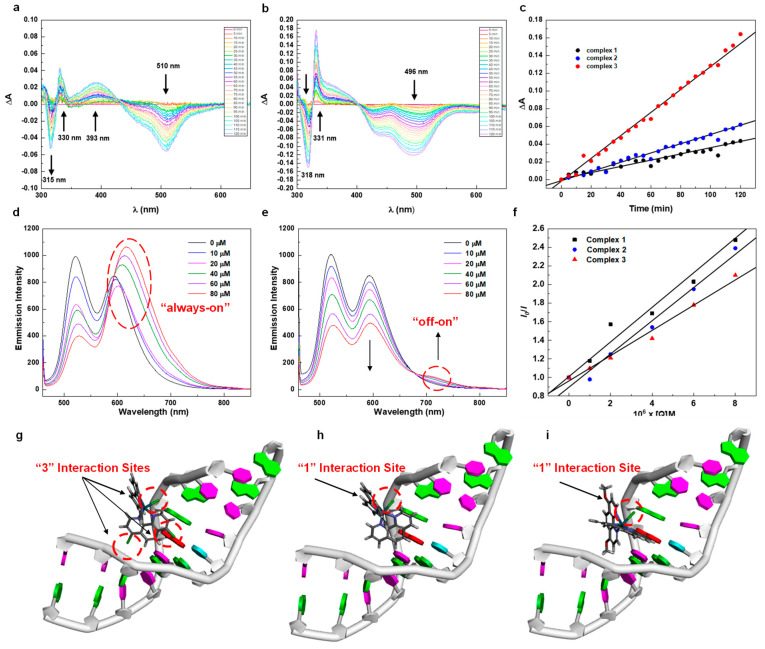
(**a**) Time evolution of UV-Vis difference spectra during the interaction of complex **1**. (**b**) Time evolution of UV-Vis difference spectra during the interaction of complex **3**. (**c**) Linear fitting of ΔA versus time. (**d**) Emission spectra of EB bound to DNA in the presence of complex **1**. (**e**) Emission spectra of EB bound to DNA in the presence of complex **3**. (**f**) Linear fitting of *I_*0*_*/*I* versus [Q]. (**g**−**i**) DS docking of Ru(II) complex **1**−**3**. (Green parts represent the guanylate).

*L*-Histidine (*L*-His) was a typical residue of certain plasma proteins such as human serum albumin (HSA) that could energetically participate in the strong associations with Ru (II) complexes as a nucleophile, resulting in antitumor bioactivity [33]. Incubation of Ru (II) complex with *L*-His resulting in adduct formation has already been studied wildly by UV kinetics curves and also ^1^H NMR spectrum. In order to check the lifetime of these complexes, we checked the stability of these complexes by checking the ^1^H NMR spectroscopy in DMSO-*d*_6_ over 30 days, finding out that they were very stable as illustrated in Figures S13–S15. Then, we recorded the ^1^H NMR spectrum of complexes **1**–**3** and *L*-His mixed together during various periods as shown in Figure S16, the peaks are clear and sharp at the beginning, indicating that there is no interaction happening between complexes **1**–**3** and *L*-His after 10 min. However, all the proton peaks became broad and blurred at 3 h, illustrating there were moderate interactions between *L*-His and complexes. Interestingly, in the next 72 h, decomposition of the adduct occurred gradually involving the release of the free ligand, as the formation of multiple clear and sharp signals was observed again, and the system reached an equilibrium between the free ligand and *L*-His. Importantly, it was worth noting that the reaction rates of these three different complexes were different and could be reflected in the ^1^H NMR spectrum, especially at 48 h. Additionally, we checked the time evolution of UV-Vis difference spectra during the interaction of the complexes **1**–**3** with *L*-His in 25 mM Hepes buffer containing 30 mM NaCl (pH 7.4) at 310 K. It is obvious that their slopes (*k*) of change over time represent the reaction rate of complexes directly, which are increasing with the enhancement of electron-donating abilities by the substituents, among which complex **3** owns the largest *k*_3_ of 1.33 × 10^−3^ compared with complex **2** (*k*_2_ of 0.50 × 10^−3^) and complex **1** (*k*_1_ of 0.31 × 10^−3^) as depicted in Figure 3a–c and Figure S17. Furthermore, we checked the mass spectrum of the mixture of *L*-His and complexes after the experiment by MALDI-TOF, finding the clear target peaks of the adducts of these two types of compounds. Such results are direct proof that the binding reaction happened. Interestingly, all of the adducts showed the target peaks minus two, which may be attributed to the oxidation of *L*-His from amines to imines by the Ru complexes as illustrated in Figure S18 [34]. These results are in line with DFT calculations, the time-depended ^1^H NMR spectrum studies between complexes and *L*-His (ratio of 1:3). Accordingly, increasing the electron-donating ability of the ligands would benefit the interaction with *L*-His, resulting in better cytotoxicity which we have already claimed before. 

Besides the interaction with *L*-His, DNA-binding properties of Ru (II) complexes were also significant for the metallodrugs [35]. Basically, there are two binding modes of Ru (II) complexes with DNA, including covalent and noncovalent interactions. The covalent binding mainly contributed to the substitution of a leaving group (-Cl) in the complex by amino fragments of DNA such as guanine (N_7_). In addition, noncovalent binding was referred to as intercalation, electrostatic, or groove bindings, which mainly relied on the electronic structures of Ru (II) complexes. The bindings of the DNA are often associated with structure distortions and then anticancer activity [36]. Hence, understanding the relationship between electronic structures and DNA-binding properties was a crucial pathway for revealing the mechanism of anticancer agents. Based on these, we next carried out fluorescence quenching studies with DNA and ethidium bromide (EB) to investigate the relationship between electronic structures and DNA interaction effects. The linear Stern–Volmer equation was an efficient indicator to reflect the rates of EB displacement from DNA by complexes **1**–**3**. The constants of Stern–Volmer equation values *(K*_sv_) of quenching were determined to be 2.0 × 10^4^, 1.8 × 10^4^, and 1.7 × 10^4^ M^−1^ for compounds **1**–**3**, respectively. Remarkably, as we mentioned before, only complex **1** showed significant luminescence emission (675 nm) due to the well HOMO-LUMO separation distribution, and such a result also corresponded with the fluorescence quenching curves as shown in Figure 3d–f and Figure S19. Surprisingly, the DNA-binding ability was not enhanced with the increase in the electron-donating ability as we expected, since stronger coordination of a Lewis base to a less electron-rich metal center is expected, and the release of the chloride ligand is not contradictory to this aspect. Further, to fully understand the relationship between electronic structure and DNA-binding effect, we preformed the molecular docking study through *Discovery Studio 2020* as shown in Figure 3g–i. Notably, the DNA-binding ability was not only related to the substitution of Cl^−^ by guanine (N_7_), but also the binding pose of Ru(II) complexes. Though increasing the ability of electron donors would make the Cl^−^ easy to leave and increase the interaction between complexes with DNA, the binding pose was also nonnegligible, which would influence the electronic-binding effects such as intercalation and groove binding even with similar structures. As for complex **1**, in addition to the labile Cl^−^ towards the green-colored guanines (N7), the suitable pose of ligand (4,7-dichloro-1,10-phenanthroline) was also directed to the other two guanines (N_7_), respectively, with the least binding energy (−185.0 kcal/mol). In addition, for complex **2**, the binding energy was −180.3 kcal/mol, which may be due to the less steric hindrance with smaller substituents (-H) leading to the flexible binding pose. Finally, the binding energy was −179.6 kcal/mol for complex **3**, which was mainly attributed to the substitution of labile Cl (covalent binding) and the electronic-binding effect of terpyridine (noncovalent binding). Interestingly, the smallest electronic-binding pose binding energy (i.e., the interaction activated energy between complex and DNA) for complex **1** was mainly attributed to the other two “guanine (N_7_)→Cl” interactions of the 1,10-phenanthroline ring. Accordingly, the interaction between Ru(II) complexes and DNA should not only consider the substitution of “Ru-Cl”, but also the electronic binding pose. For example, the electron-donating substituents of complex **3** posed outside the DNA, while the electron-withdrawing substituents of complex **1** was directed to the guanines (N_7_).

### 2.4. Cell Cytotoxicity and Bioimaging

**Figure 4:**
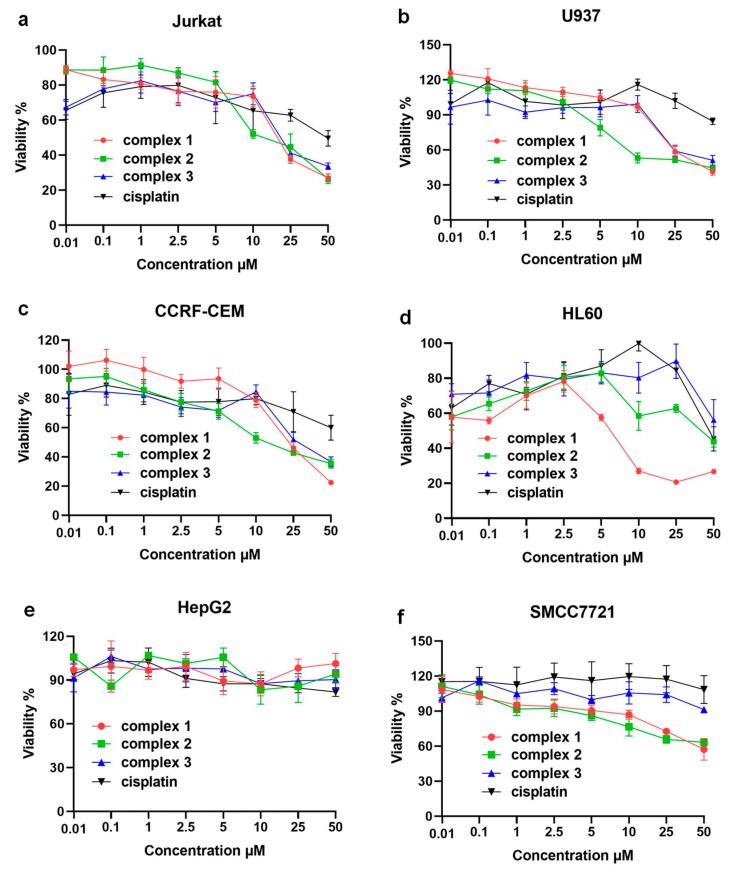
Plot of viable cells at increasing concentrations of complex **1**–**3** against: (**a**) Jurkat, (**b**) U937, (**c**) CCRF-CEM, (**d**) HL60, (**e**) HepG2, and (**f**) SMCC7721 tumor cells.

Further, in order to study the relationship between electronic structures and bioactivities of these complexes, we assayed the cytotoxicity of complexes **1**–**3** in the immortalized T lymphocyte cell line (Jurkat), promyelocytic cell line derived from human leukemia (HL60), a human myelomonocytic tumor cell line (U937), a human T lymphoblasts tumor cell line (CCRF-CEM), and two human hepatoellular carcinomas cell lines (HepG2 and SMCC7721) by the 3-(4,5)-dimethylthiahiazo(-z-y1)-3,5-di-phenytetrazoliumromide (MTT) method and the results were compared using cell viability curves with a concentration range from 0.01 μM to 50.0 μM as depicted in Figure 4a–f. The results showed that these compounds specifically inhibited the proliferation of hematological tumor cells, but had a weaker inhibitory effect on the proliferation of solid tumor hepatocellular carcinoma cells, with IC_50_ values all greater than 50 μM (specific IC_50_ values are shown in Table S1). The comparison of cytotoxicity results showed that the Ru(II) complexes had a stronger inhibitory effect on four types of hematological tumor cells than cisplatin. The three complexes showed similar effects against Jurkat, CCRF-CEM and U937, but for HL60, the inhibitory effect of complex **1** (IC_50_ values of 6.23 ± 0.26 μM) was significantly better than that of complex **2** (IC_50_ values of 42.15 ± 2.22 μM) and **3** (IC_50_ values over 50 μM). Interestingly, the MTT results were not in line with traditional expectations because complex **3** with electron-donating groups should own a faster hydrolysis process than the other two complexes, which generally results in better bioactivity against the tumor cells. However, such results corresponded to the DNA interaction studies in which complex **1** owned the best interaction performance (*K*_sv_ = 2.0 × 10^4^ M^−1^) and the least DNA binding energy (−185.0 kcal/mol). Furthremore, the cytotoxicity of these kinds of complexes also depended on the treated cell lines, which were more sensitive to the DNA/RNA-induced pathways such as HL60 itself. HL60 cells could regulate the release of their own DNA and enhance the negative charge on the surface of the cell membrane [37,38]. More importantly, the design strategy of optimizing the electron donating or electron withdrawing should not only consider the substitution rate of the labile leaving group but also electronic binding abilities. Such a conclusion was a supplemental guideline for developing N, N- polydentate type Ru (II)-based anticancer drugs.

**Figure 5:**
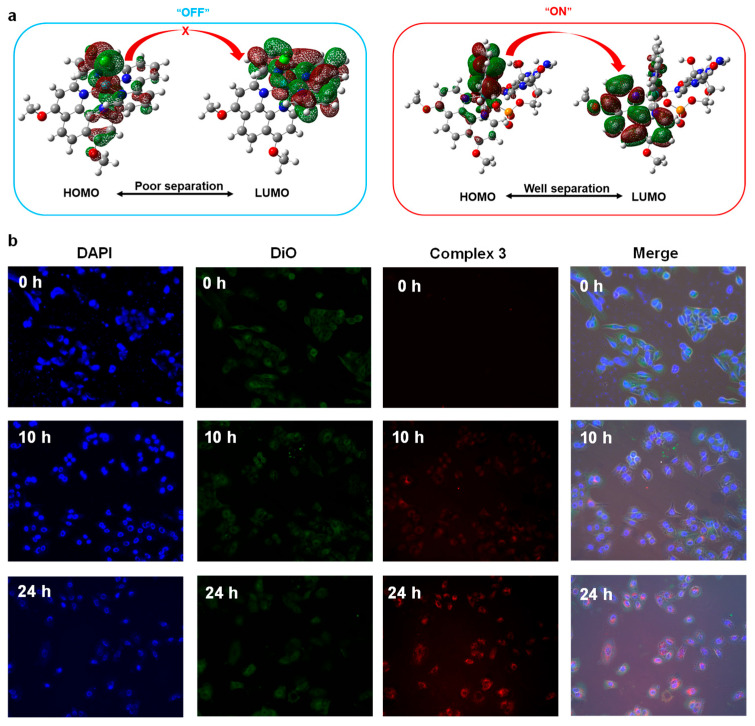
(**a**) The mechanism for the “off-on” signature for complex **3**, (**b**) Time various cellular imaging contrast of A549 cells by DAPI, DiO, and complex **3**.

Further, considering the DNA-binding activity and the “off-on” feature as potential bioimaging probes of electron-donating Ru (II) complexes, we further studied the cellular imaging property of Ru (II) complex **3**. We selected the blue emitter, 4′,6-diamidino-2-phenylindole (DAPI), as a standard DNA-specific probe that could generate blue fluorescence in 460 nm by attaching in the minor grove of A-T rich sequences of DNA. In addition, we selected the green fluorescent lipophilic tracer emitter, 3,3′-dioctadecyloxacarbocyanine perchlorate (DiO), as a comparison probe. To verify that the distribution altering of HOMO and LUMO orbital (before/after interaction with guanines) would result in an “off-on” signature of Ru(II) complexes with electron-donating groups (complex **3**) as illustrated in Figure 5a, we stained the A549 cells with metallodrugs complex **3** along with the DAPI and DiO to evaluate the DNA binding and bioimaging abilities at various times (0 h, 10 h, 24 h) as shown in Figure 5b. The results showed that the brightness of the red fluorescence enhanced with the increasing interaction time and the dyed target part for complex **3** coincided with the blue fluorescence of DAPI (nuclear staining) instead of DiO (lipophilic staining). This time-dependent fluorescence indicated that complex **3** was DNA targeting and owned an “off-on” light switching signature, which could be attributed to the change of “HOMO-LUMO” distribution via the substitution of labile Cl. Such an impressive result could also be used as a latent fluorescence probe by simplifying the response linker as a Cl atom to detect capacity live-cell delivery methods for other biomedically important issues.

## 3. Materials and Methods

### 3.1. General Information

Ruthenium(III) chloride trihydrate, terpyridine, and 1,10-phenanthroline derivatives were all purchased from Alfachem Technology Co. Ltd. (Zhengzhou City, China). ^1^H NMR spectra were measured on a Bruker-500 MHz/400 MHz spectrometer, and ^13^C NMR was measured on a Bruker-125 MHz spectrometer. Mass spectra were recorded on a MALDI-TOF spectrometer. Ultraviolet-visible absorption spectra were recorded by Shimadzu UV-2600 spectrophotometer. The PL spectrums were recorded by Hitachi fluorescence spectrophotometer F-7100.

### 3.2. Synthesis and Characterization of [Ru(tpy)(R-phen)Cl](PF_6_)

**Scheme 1:**
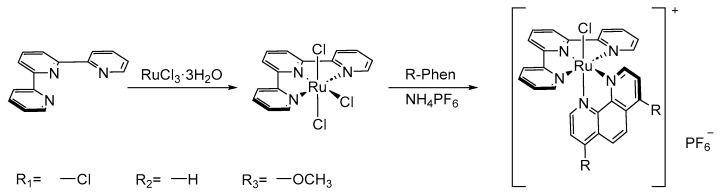
Synthesis route of complexes **1**–**3**.

The synthetic procedure was according to the literature in previous work [39,40,41]. A mixture of R-phen (0.5 mmol), Ru(tpy)Cl_3_ (220 mg, 0.5 mmol), and LiCl (105.4 mg 2.5 mmol) in EtOH: H_2_O (3:1, 50 mL) with Et_3_N (3 drops) was refluxed for 12 h. After that, the dark red reaction mixture was cooled to room temperature, then concentrated in vacuo. Then, NH_4_PF_6_ (238.1 mg. 2.1 mmol) in water (100 mL) was added to the mixture and stirred for 5 min. After that, the insoluble materials were filtered and washed with water, and ether, respectively. The resulting residues were purified by CH_3_CN: toluene (1:1) on Al_2_O_3_ (50 g) through chromatography to afford the desired complexes as shown in Scheme 1.

[Ru(tpy)(Cl-phen)Cl](PF_6_) (**1**) (76%) *R*_f_ = 0.57 [toluene:CH_3_CN (1:1)]:

^1^H NMR (500 MHz, CD_3_CN, δ): 10.38 (d, *J* = 6.8 Hz, 1H, Ar-H), 8.65 (d, *J* = 11.1 Hz, 1H, Ar-H), 8.53 (d, *J* = 9.7 Hz, 2H, Ar-H), 8.42–8.45 (m, 2H, Ar-H), 8.38 (d, *J* = 9.7 Hz, 2H, Ar-H), 8.15 (t, *J* = 9.6 Hz, 1H, Ar-H), 7.85 (t, *J* = 9.4 Hz, 2H, Ar-H), 7.65 (d, *J* = 7.0 Hz, 1H, Ar-H), 7.53 (d, *J* = 6.6 Hz, 2H, Ar-H), 7.42 (d, *J* = 7.0 Hz, 1H, Ar-H),7.14 (t, *J* = 8.0 Hz, 2H, Ar-H). ^13^C NMR (CD_3_CN, 125 MHz, δ) 159.44, 158.90, 154.67, 154.30, 153.60, 150.89, 149.19, 142.54, 141.47, 138.13, 135.17, 130.18, 129.84, 128.00, 127.47, 126.33, 125.96 125.66, 124.49, 123.52. Elemental analysis: calcd (%) for C_29_H_23_ClF_6_N_5_O_2_PRu (MW: 763.85):C, 42.46; H, 2.24; N, 9.17%. Found: C, 42.34; H, 2.28; N, 9.25%. MS (MALDI-TOF) *m*/*z*: [M]^+^ calcd for C_27_H_17_Cl_3_N_5_Ru, 619.96; found, 619.93 (as shown in Figures S1–S3).

[Ru(tpy)(phen)Cl](PF_6_) (2) (55%) *R*_f_ = 0.48 [toluene:CH_3_CN (1:1)]:

^1^H NMR (500 MHz, CD_3_CN, δ): 10.43 (dd, *J* = 1.4 Hz, 5.2 Hz, 1H, Ar-H), 8.81 (dd, *J* = 1.3 Hz, 5.6 Hz, 1H, Ar-H), 8.52 (d, *J* = 8.2 Hz, 2H, Ar-H), 8.38 (dt, *J* = 1.1 Hz, 8.1 Hz, 2H, Ar-H), 8.29–8.33 (m, 2H, Ar-H), 8.22 (dd, *J* = 1.2 Hz, 8.1 Hz, 2H, Ar-H), 8.12 (t, *J* = 8.1 Hz, 1H, Ar-H), 8.08 (d, *J* = 8.9 Hz, 1H, Ar-H), 7.83 (td, *J* = 1.6 Hz, 8.1 Hz, 2H, Ar-H), 7.66 (dd, *J* = 1.2 Hz, 5.4 Hz, 1H, Ar-H), 7.50–7.52 (m, 2H, Ar-H), 7.29 (dd, *J* = 2.8 Hz, 8.1 Hz, 1H, Ar-H), 7.11–7.14 (m, 2H, Ar-H). ^13^C NMR (CD_3_CN, 125 MHz, δ) 159.60, 159.15, 154.03, 153.66, 153.41, 150.06, 148.31, 137.85, 136.32, 135.35, 134.59, 131.75, 131.21, 128.83, 128.38, 127.98, 126.79, 125.68, 124.37, 123.37. Elemental analysis: calcd (%) for C_27_H_19_ClF_6_N_5_PRu (MW: 694.97):C, 46.66; H, 2.76; N, 10.08%. Found: C, 46.89; H, 2.69; N, 10.11%. MS (MALDI-TOF) *m*/*z*: [M]^+^ calcd for C_27_H_19_ClN_5_Ru, 550.04; found, 550.02 (as shown in Figures S4–S6).

[Ru(tpy)(MeO-phen)Cl](PF_6_) (**3**) (83%) *R*_f_ = 0.55 [toluene:CH_3_CN (1:1)]:

^1^H NMR (500 MHz, CD_3_CN, δ): 10.15 (d, *J* = 6.1 Hz, 1H, Ar-H), 8.49 (d, *J* = 8.1 Hz, 2H, Ar-H), 8.43 (d, *J* = 9.1 Hz, 1H, Ar-H), 8.36 (d, *J* = 8.0 Hz, 2H, Ar-H), 8.20 (d, *J* = 9.0 Hz, 1H, Ar-H), 8.05 (t, *J* = 8.0 Hz, 1H, Ar-H), 7.79–7.83 (m, 2H, Ar-H), 7.63 (d, *J* = 5.4 Hz, 2H, Ar-H), 7.33 (d, *J* = 6.1 Hz, 1H, Ar-H), 7.14–7.16 (m, 2H, Ar-H), 6.73 (d, *J* = 6.1 Hz, 1H, Ar-H), 4.36 (s, 3 H; O-CH_3_), 3.93 (s, 3 H; O-CH_3_). ^13^C NMR (CD_3_CN, 125 MHz, δ) 163.61, 162.72, 159.92, 159.85, 154.95, 154.26, 153.16, 149.59, 148.37, 137.39, 133.49, 127.88, 124.14, 123.92, 123.56, 123.11, 121.76 121.24, 107.41, 106.49, 59.04, 57.68. Elemental analysis: calcd (%) for C_29_H_23_ClF_6_N_5_O_2_PRu (MW: 755.02):C, 46.13; H, 3.07; N, 9.28%. Found: C, 46.16; H, 3.10; N, 9.33%. MS (MALDI-TOF) *m*/*z*: [M]^+^ calcd for C_29_H_23_ClN_5_O_2_Ru, 610.06; found, 610.13 (as shown in Figures S7–S9) [27].

### 3.3. Theoretical Calculations

The electronic properties and energy levels of targeted Ru(II) complexes were calculated by the Gaussian 16 program package. B3LYP (Becke three parameters hybrid functional with Lee–Yang–Perdew correlation functionals) was used as the calculation model with the 6–31G(d) atomic and LANL2DZ basis according to the general choice for these kind of complexes [42]. The molecular orbitals were visualized by Gaussview 6.0 program.

Molecular docking studies were performed through Discovery Studio 2020 software. All the ligands were picked up from the optimized structures by the Gaussian 16 program and the crystal of DNA (PDB: 6g8s) was downloaded from the RCSB PDB protein data bank. The target DNA was prepared by deleting the original ligands, water, and heteroatoms. The CDOCKER procedure was used to figure out the binding poses and energies of Ru complexes **1**–**3** with DNA (6g8s).

### 3.4. DNA Binding Studies

DNA-binding effects were investigated by fluorescence quenching studies with ethidium bromide (EB) and DNA. Stock solutions of ct-DNA (2.0 mM) and ruthenium complexes (0.1 mM) were mixed in a 10 mM Tris-HCl buffer solution (pH = 7.6, 150 mM NaCl). The complex–DNA solutions were incubated and shaken for 10 min at room temperature by mixing DNA solutions with various concentrations of the complexes, which varied from 0.0 μM, 10 μM, 20 μM, 40 μM, 60 μM, to 80.0 μM. The luminescent emission intensities were recorded with the excitation wavelength set at 433 nm (slit widths 10 nm) and the luminescent emission set at 460 nm (slit widths 20 nm).

### 3.5. L-Histidine Interaction Studies

The Ultraviolet-visible (UV-vis) spectroscopy is used to obtain the absorbance spectra of the reactions initiated by mixing a solution of each complex (20 μL, 1.00 mM) with 180 μL L-His (4.45 mM) in a 96-well plate containing 25 mM Hepes buffer and 30 mM NaCl at pH 7.40. At 310 K, the SpectraMax Paradigm multi-function microplate reader was used to read the spectrum of 230–1000 nm wavelengths in ABS read mode within 2 h, intervals of 5 min, shaking 5 s before each read (Δ*A* = *A_t_* − *A*_0_, where *A_t_* = absorbance at time *t* and *A*_0_ = absorbance at the time at which the first spectrum was recorded, 330 nm for complexes **1**–**2**, and 331 nm for complex **3**). Time-depended ^1^H NMR spectrum (aromatic part) of complexes with *L*-histidine was recorded by Bruker-400 MHz spectrometer in DMSO-*d_6_* solvent with the ratio of 1:3 for complex and L-histidine, respectively, at 10 min, 3 h, 24 h, 48 h, and 72 h.

### 3.6. Cell Cytotoxicity

Immortalized T lymphocyte cell line (Jurkat), a promyelocytic cell line derived from human leukemia (HL60), a human myelomonocytic tumor cell line (U937), and a human T lymphoblasts tumor cell line (CCRF-CEM) was cultured in RPMI-1640-medium, and two human hepatocellular carcinomas cell lines (HepG2 and SMCC7721) were cultured in DMEM medium, with 10% fetal bovine serum (FBS) in humidified air at 37 °C with 5% CO_2_. Then these tumor cells were seeded into 96-well plates at 6–10 × 10^3^ cells/well, treated with the complex **1**–**3** at different concentrations after these adherent cells hatched for 12 h at 37 °C with 5% CO_2_. (The final concentrations of these compounds were 25, 10, 5, 2.5, 1, 0.1, and 0.01 μM). After 72 h treatment, the cells were incubated with 15 μL MTT solution (5 mg/mL) for 4 h at 37 °C with 5% CO_2_. The formazan precipitates were dissolved in 100 mL DMSO. At 490 nm, the absorbance was measured by Infinite M1000 PRO (TECAN). The Jurkat, U937, CCRF-CEM, HL-60, and HepG2 cell lines were purchased from the National Collection of Authenticated Cell Cultures (Shanghai, China). The SMCC7721 cells were purchased from Procell Life Science & Technology Co., Ltd. (Wuhan, China).

### 3.7. Cell Imaging

For the cellular imaging studies, A549 cell lines were incubated in a 12-well plate for 24 h to adhere. After being washed with PBS (0.01 M, pH 7.4), 3.19 μM complex **3**, a certain amount of DiO (Beyotime; C1993S-3) and DAPI (Beyotime; C1005) were added to the well for incubating another 0 h, 10 h, and 24 h, respectively, under standard conditions. After washing 3 times with PBS, the antifluorescence quenching agent was added to the slides, covering the slides. We fixed the glass slides with nail polish to avoid light for the whole process. A fluorescent microscope (Axio Observer 5 with ApoTome, Jena, Germany) was conducted for measurements.

## 4. Conclusions

In this study, a series of phenanthroline-type ligands with various electronic functional groups in the para position were synthesized to afford three Ru(II) complexes, namely, [Ru(tpy)(Cl-phen)Cl](PF_6_) (**1**), [Ru(tpy)(phen)Cl](PF_6_) (**2**), and [Ru(tpy)(MeO-phen)Cl](PF_6_) (**3**). All of them have one labile Cl to be an active leaving group to lead the bioactivities. Theoretical and optical studies revealed that the electron-donating abilities of these complexes were in the order: complex **1** < complex **2** < complex **3**. The interaction with protein residue (*L*-His) of three cationic complexes clearly revealed that enhancing the electron-donating ability would increase the rate of the substituent reaction and thus benefit the bioactivity. While the DNA competitive binding studies with EB suggested that it should consider the comprehensive electronic effects instead of increasing the substitution effect with guanines (N_7_). The cytotoxicity of the three Ru (II) complexes **1**–**3** was investigated against six different tumor cells (Jurkat, HL60, U937, CCRF-CEM, HepG2, and SMCC7721) by the MTT assay. The plot of viable cells at various concentrations showed that all the complexes performed better cytotoxicity against hematological tumor cells compared to the commercial clinical drug cisplatin. In particular, complex **1** was the best cytotoxic drug against the HL60 cell line with the IC_50_ value of 6.23 ± 0.26 μM, which is promising to be a potential anticancer drug candidate. Cellular imaging studies indicated that the nucleus (DNA) was the main target, and only the complexes with electron-donating groups (such as complex **3**) would be unique potential “off-on” bioimaging agents by the changes in “HOMO-LUMO” distribution after the reaction with target molecules such as DNA, while the complex with electron-withdrawing group featured an “always-on” phenomenon. Collectively, this work systematically studied the relationship between electronic structure and bioactivities of polypyridyl Ru(II) complexes, which paved the way to design these kinds of metallodrugs and analytical bioimaging probes in future studies.

## Data Availability

We inform that all the published data were obtained and involved in this research.

## Supplementary Materials

The following supporting information can be downloaded at: https://www.mdpi.com/article/10.3390/molecules28135035/s1.
