# Supplementary material for: Investigation of the Relationship between Electronic Structures and Bioactivities of Polypyridyl Ru(II) Complexes
Source: Molecules. 2023 Jun 27;28(13):5035. doi: 10.3390/molecules28135035 (PMC10343301; doi:10.3390/molecules28135035)
Supplement: Supplementary file 1 [file molecules-28-05035-s001.zip › molecules-2466033-supplementary.pdf]

Supplementary Material

Investigation of the Relationship between Electronic Structures and Bioactivities of Polypyridyl Ru(II) Complexes

Zhiying Hou [1], Yang Lu [1,*], Bin Zhang [2], A. F. M. Motiur Rahman [3], Yufen Zhao [1], Ning Xi [1], Ning Wang [1,*] and Jinhui Wang [1,*]

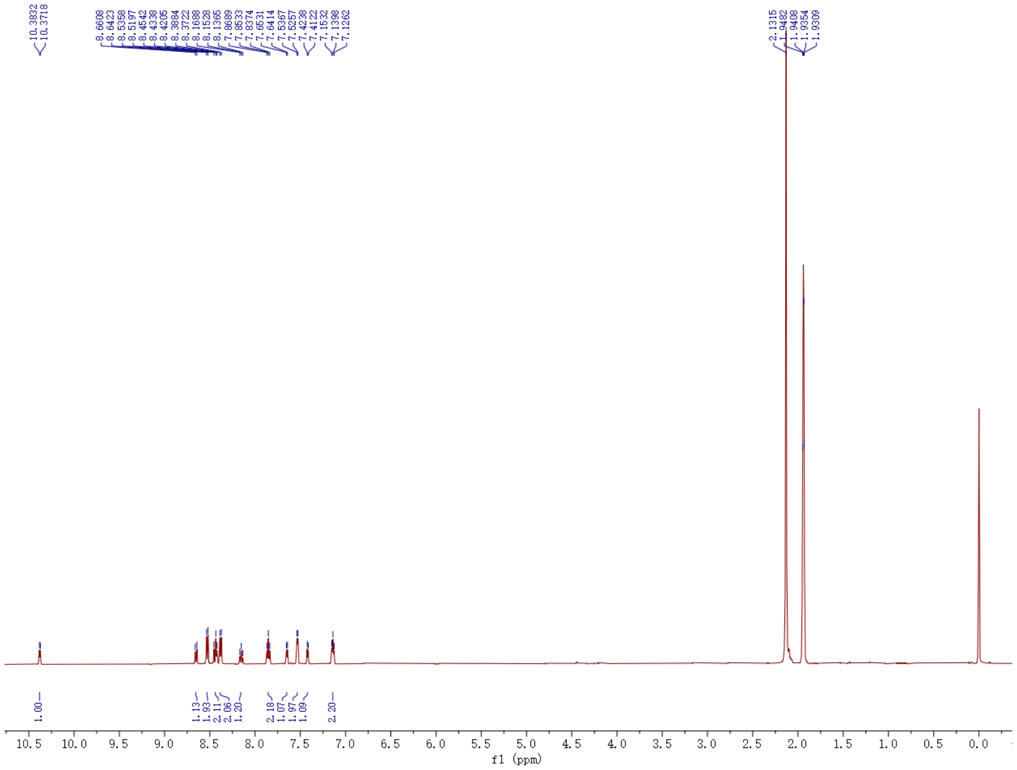

Figure S1. ^{1}H NMR spectrum (500 MHz, CD_3CN) of [Ru(tpy)(Cl-phen)Cl](PF_6) (**1**).

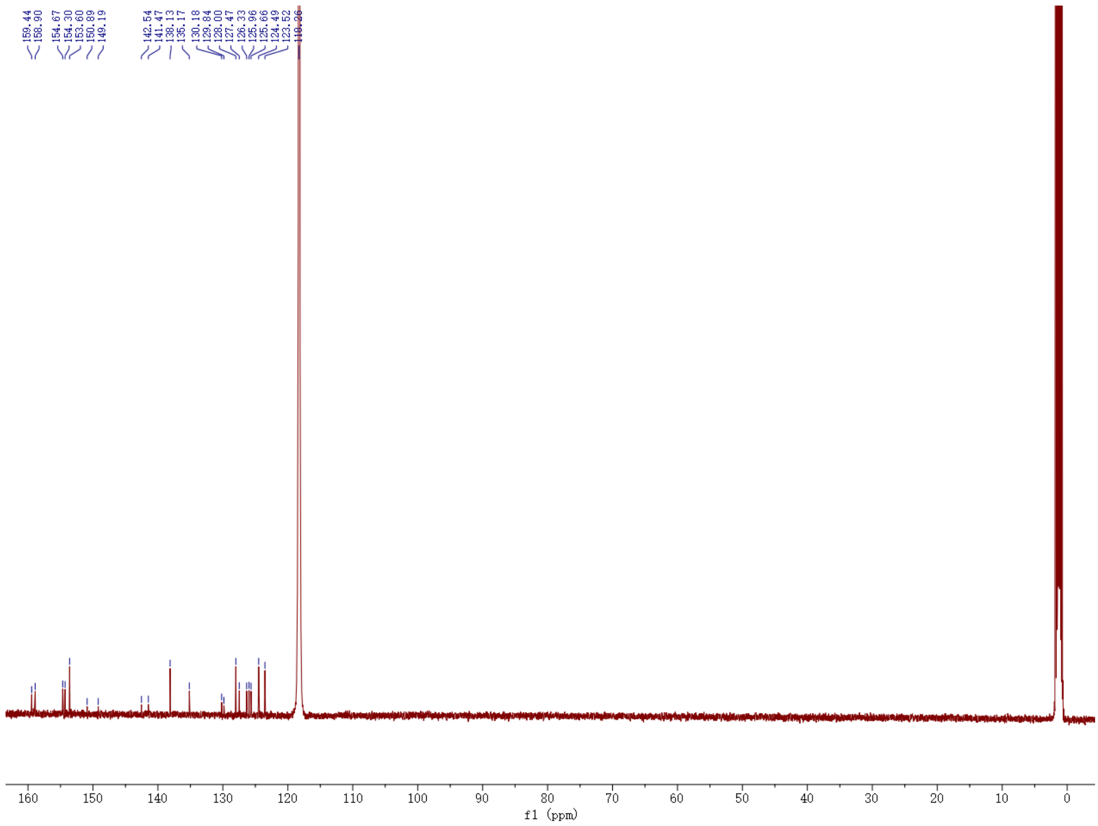

Figure S2. ^{13}C spectrum (125 MHz, CD_3CN) of [Ru(tpy)(Cl-phen)Cl](PF_6) (**1**).

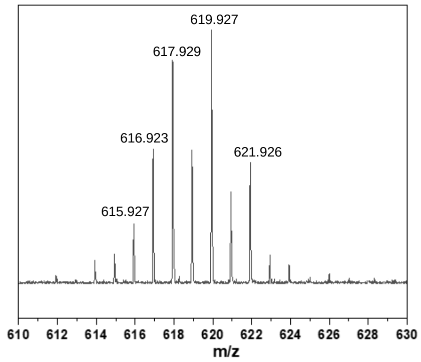

Figure S3. MALDI-TOF mass spectra of [Ru(tpy)(Cl-phen)Cl](PF_6) (**1**).

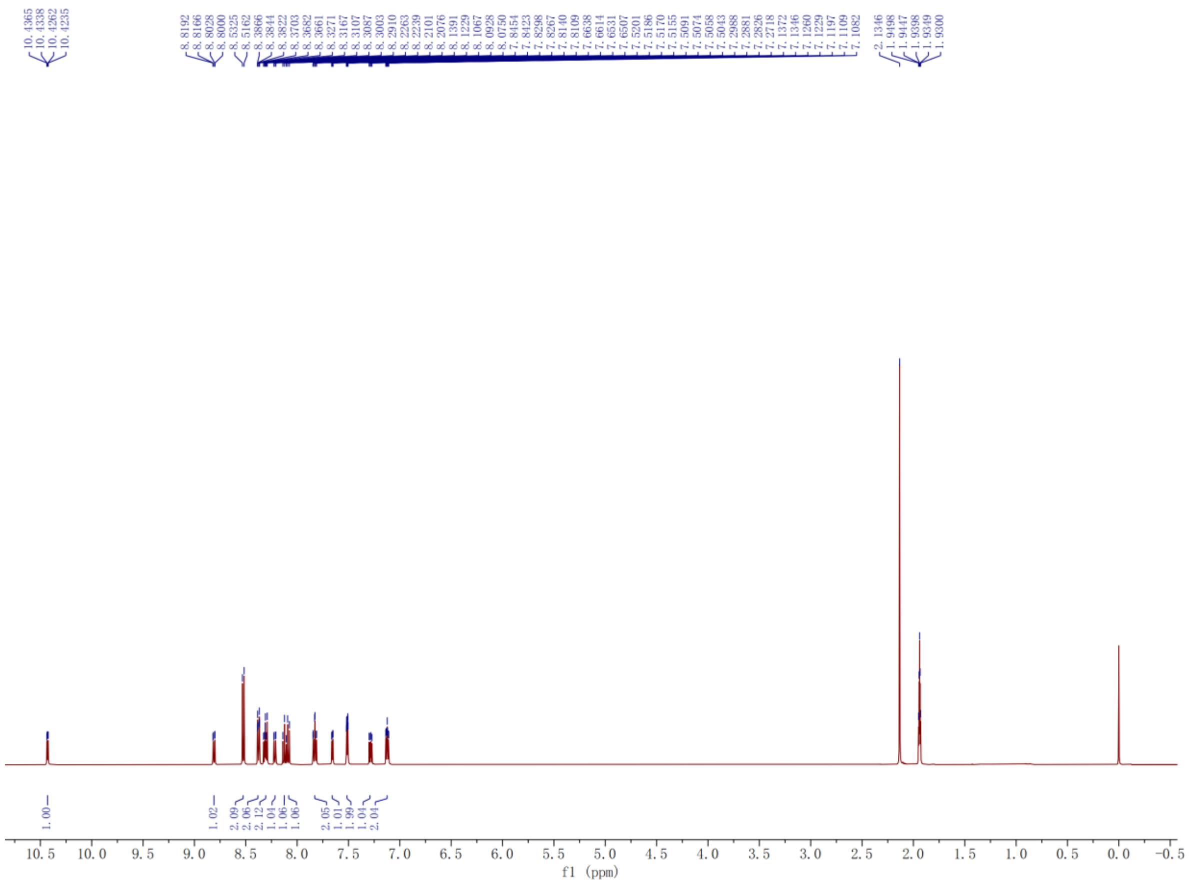

Figure S4. ^{1}H NMR spectrum (500 MHz, CD_3CN) of [Ru(tpy)(phen)Cl](PF_6) (**2**).

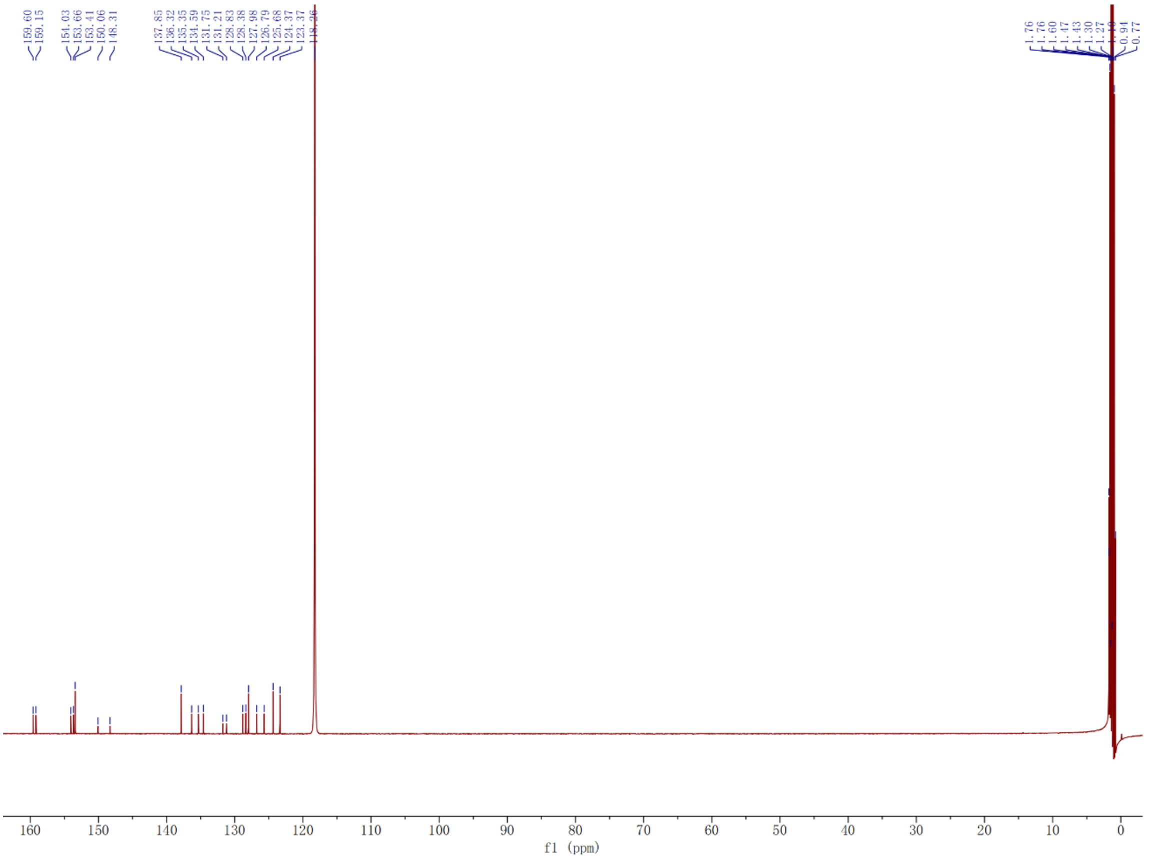

Figure S5. ^{13}C NMR spectrum (125 MHz, CD_3CN) of [Ru(tpy)(phen)Cl](PF_6) (**2**).

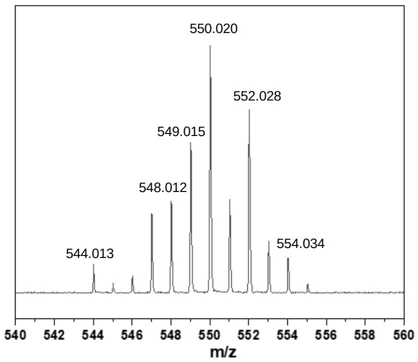

Figure S6. MALDI-TOF mass spectra of [Ru(tpy)(phen)Cl](PF_6) (**2**).

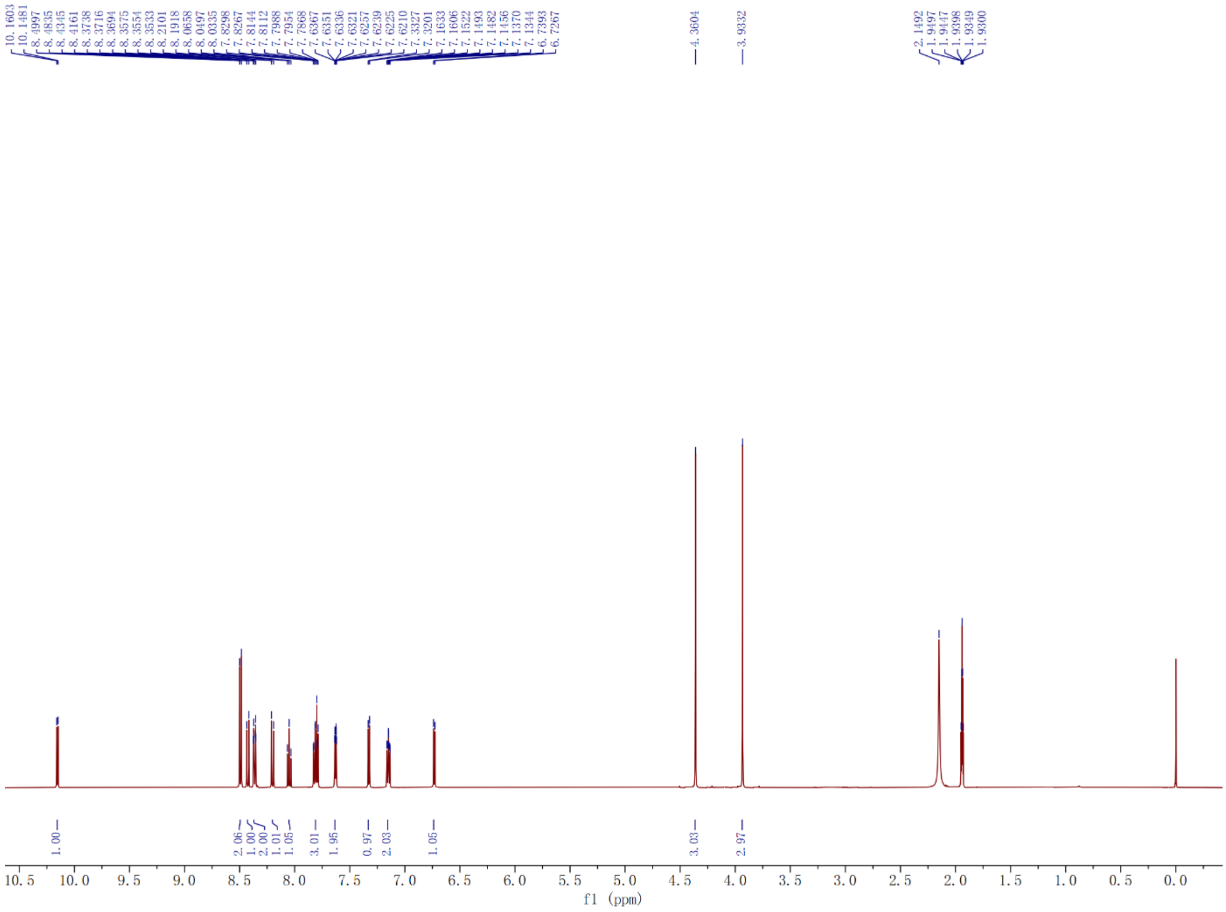

Figure S7. ^{1}H NMR spectrum (500 MHz, CD_3CN) of [Ru(tpy)(Meo-phen)Cl](PF_6) (**3**).

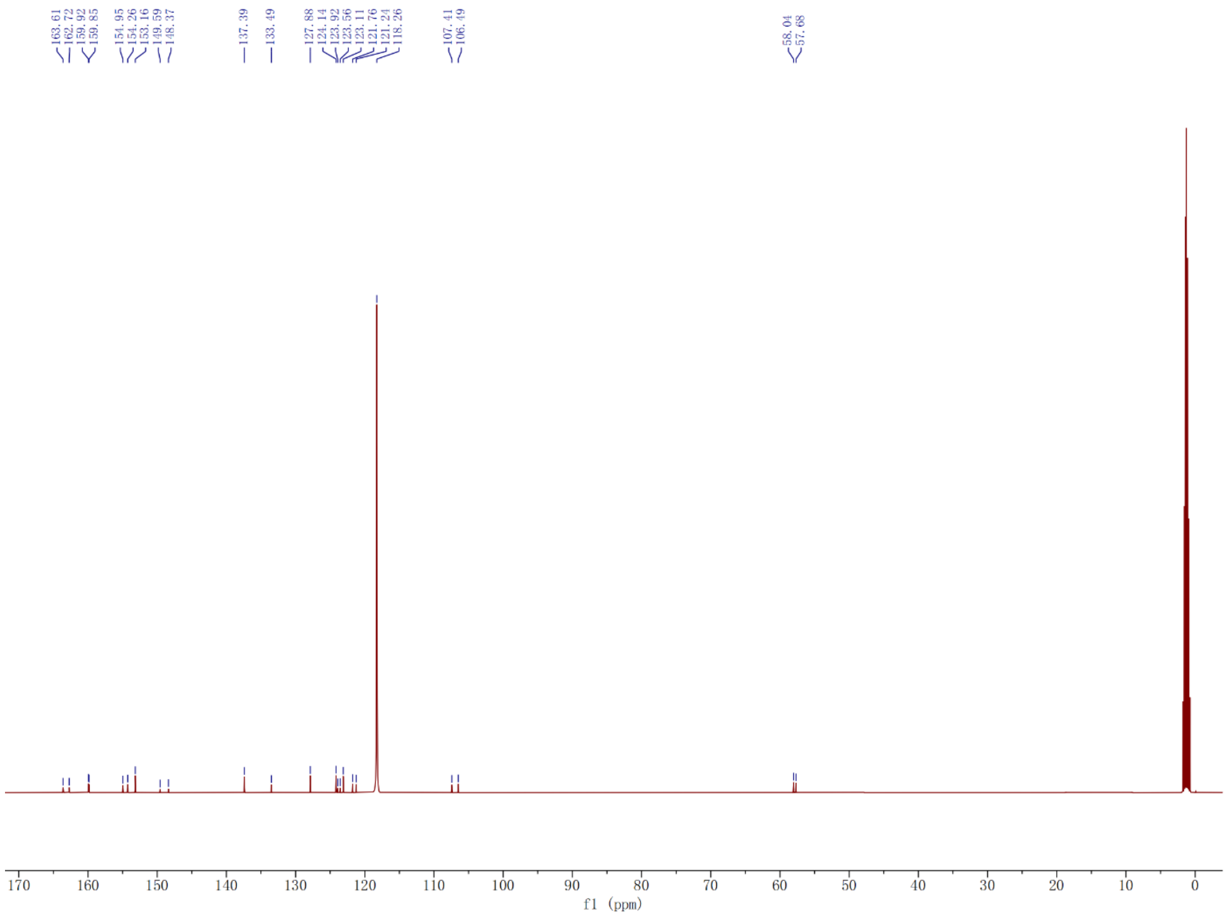

Figure S8. ^{13}C NMR spectrum (125 MHz, CD_3CN) of [Ru(tpy)(Meo-phen)Cl](PF_6) (**3**).

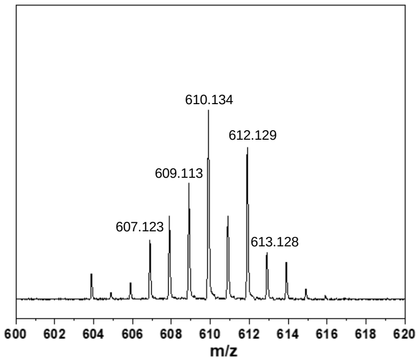

Figure S9. MALDI-TOF mass spectra of [Ru(tpy)(MeO-phen)Cl](PF_6) (**3**).

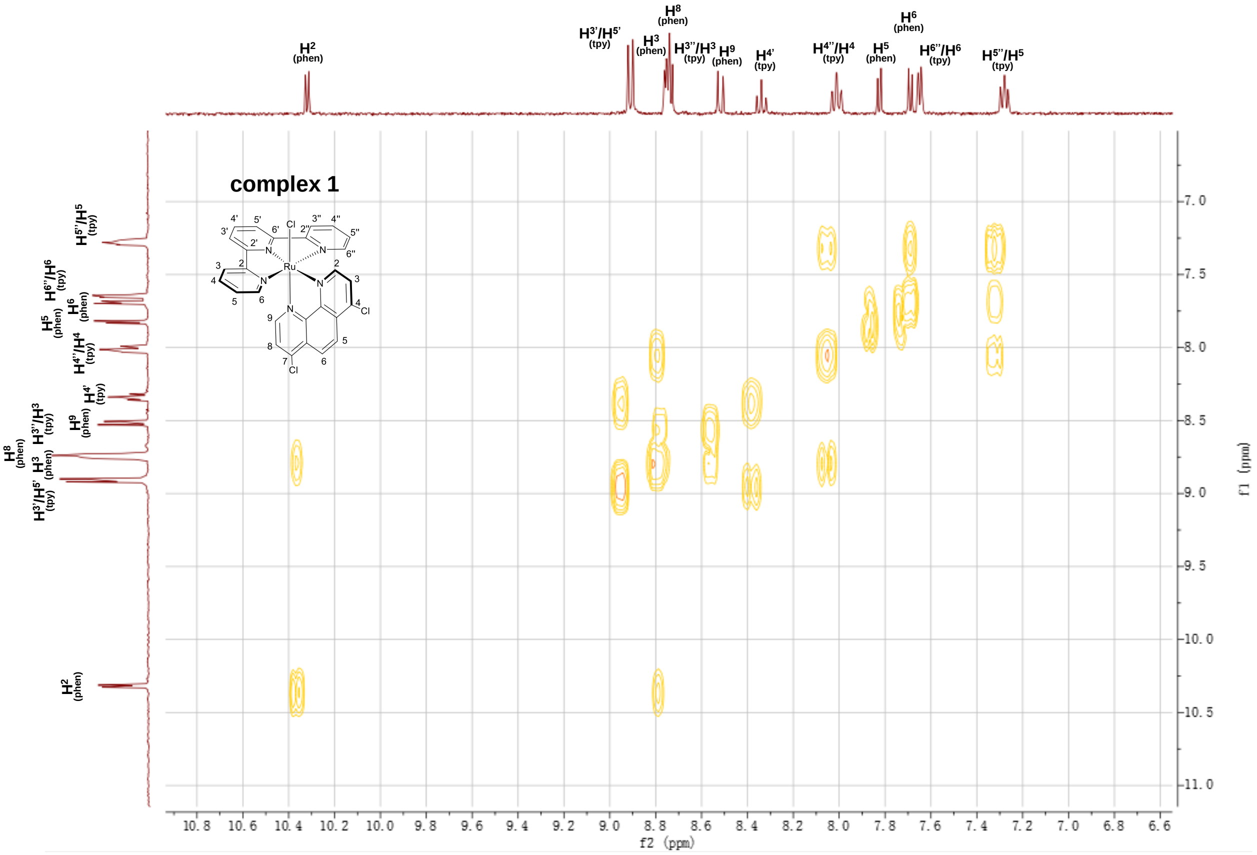

Figure S10. COSY spectrum (400 MHz, DMSO) of [Ru(tpy)(Cl-phen)Cl](PF_6) (**1**).

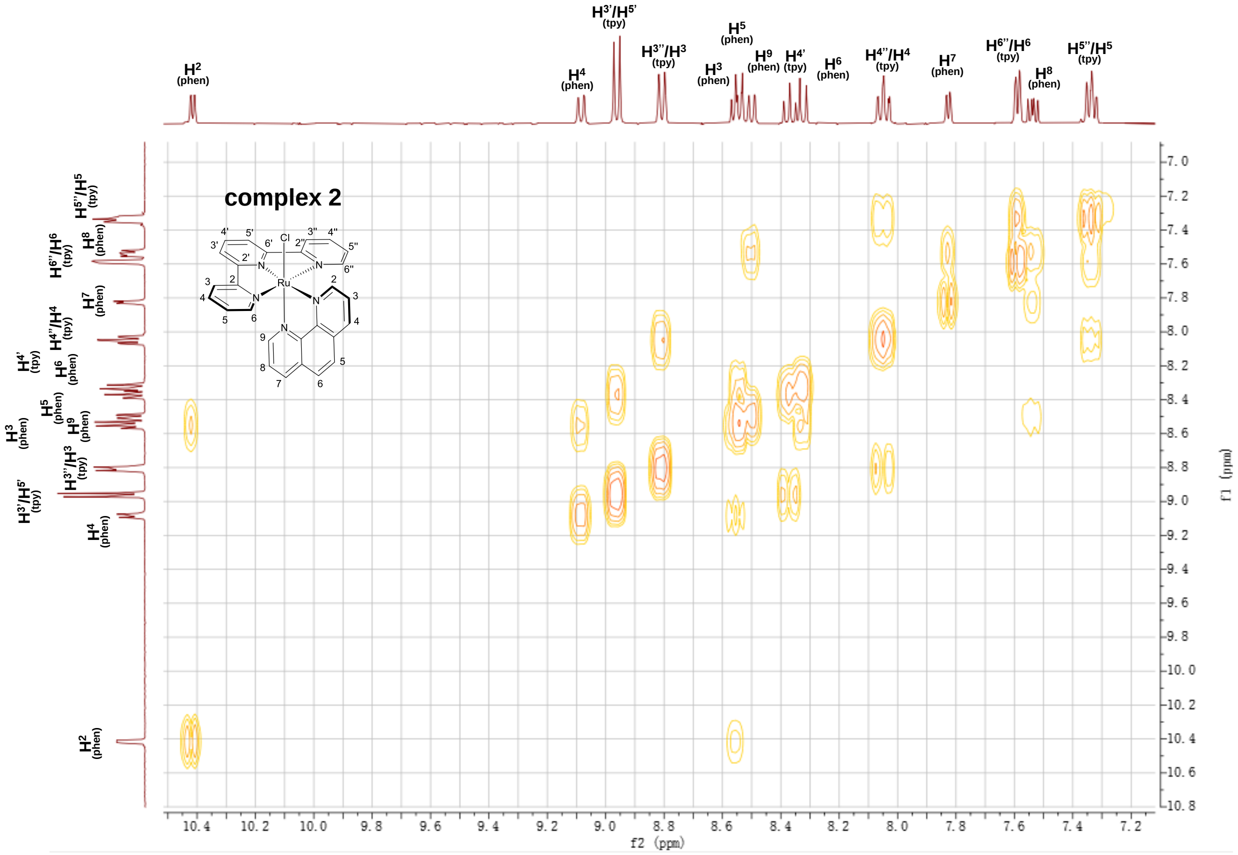

Figure S11. COSY spectrum (400 MHz, DMSO) of [Ru(tpy)(phen)Cl](PF_6) (**2**).

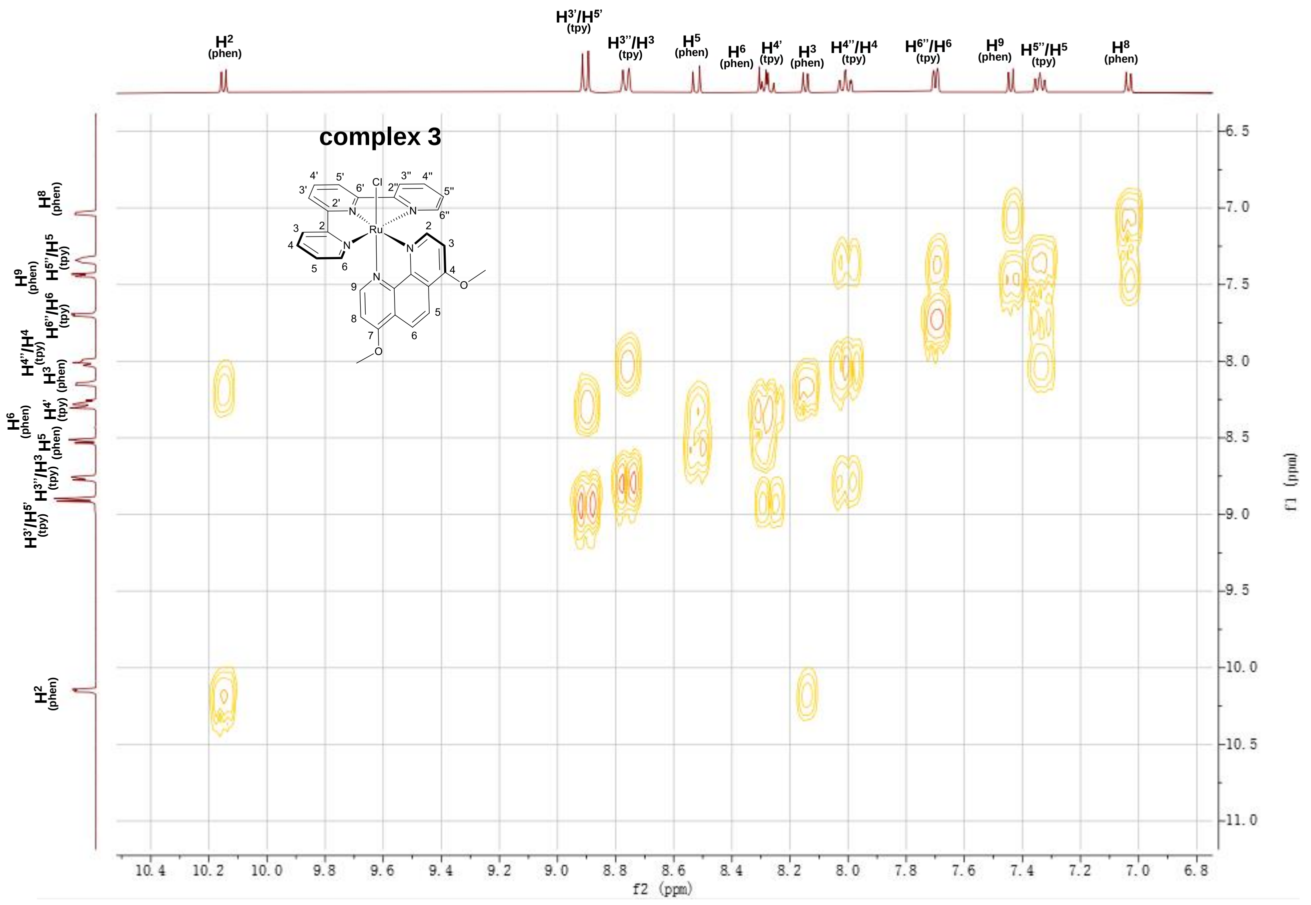

Figure S12. COSY spectrum (400 MHz, DMSO) of $[Ru(tpy)(MeO\text{-}phen)Cl](PF_6)$ (**3**).

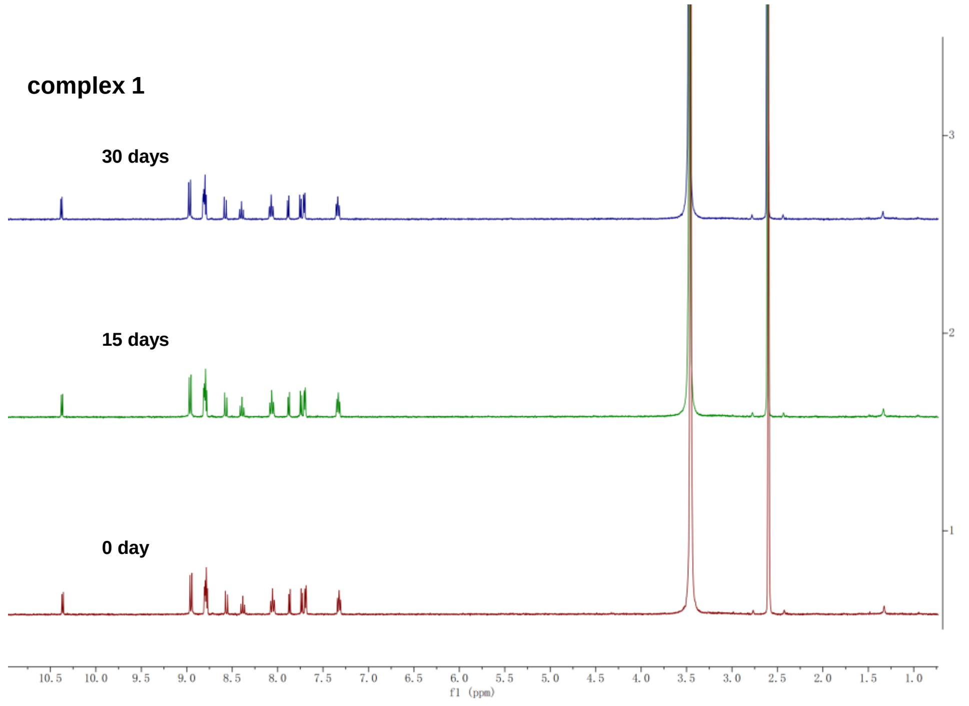

Figure S13. The stability of complex **1** was checked by ^{1}H NMR spectrum over 30 days.

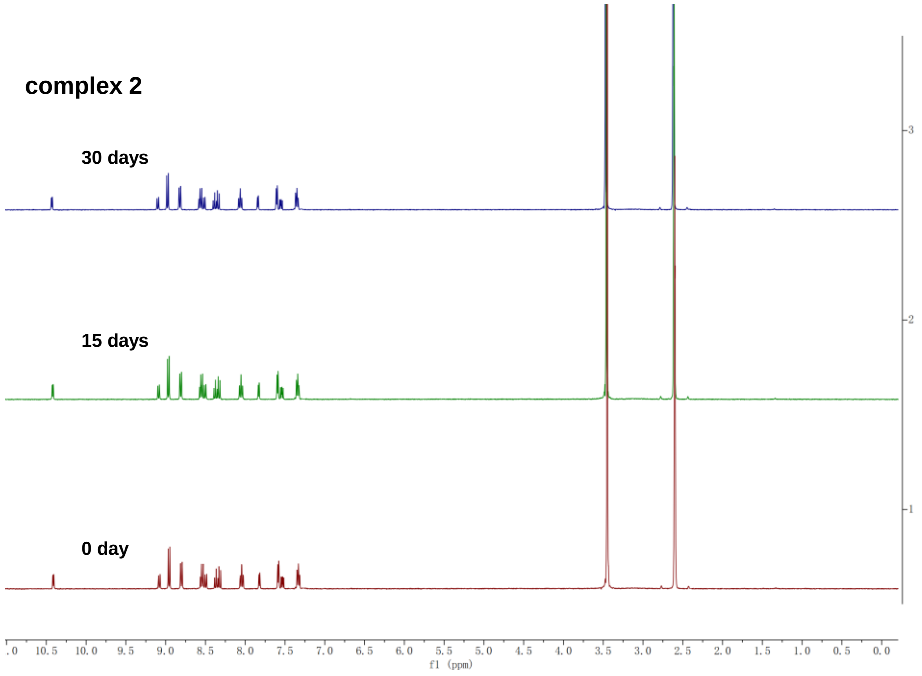

Figure S14. The stability of complex **2** was checked by ^{1}H NMR spectrum over 30 days.

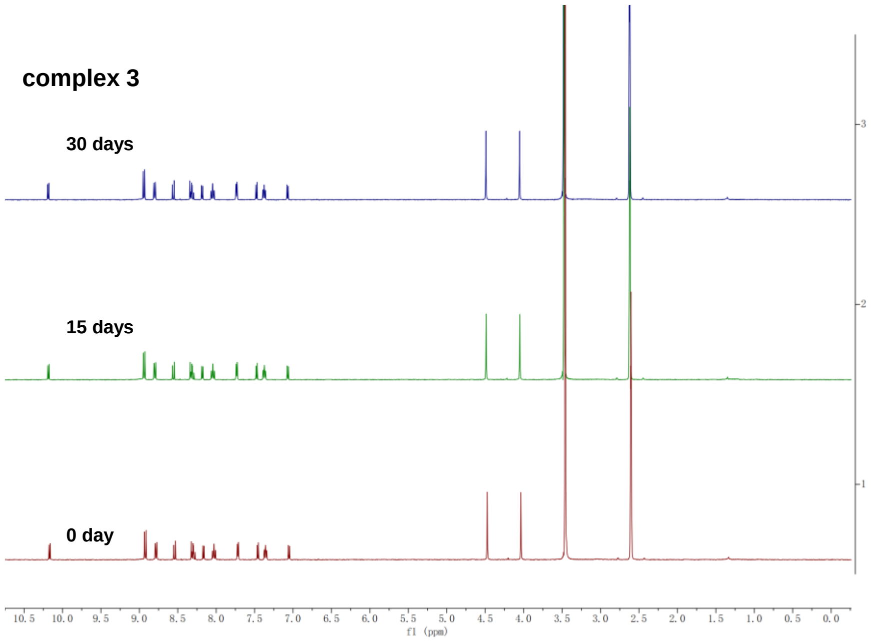

Figure S15. The stability of complex **3** was checked by ^{1}H NMR spectrum over 30 days.

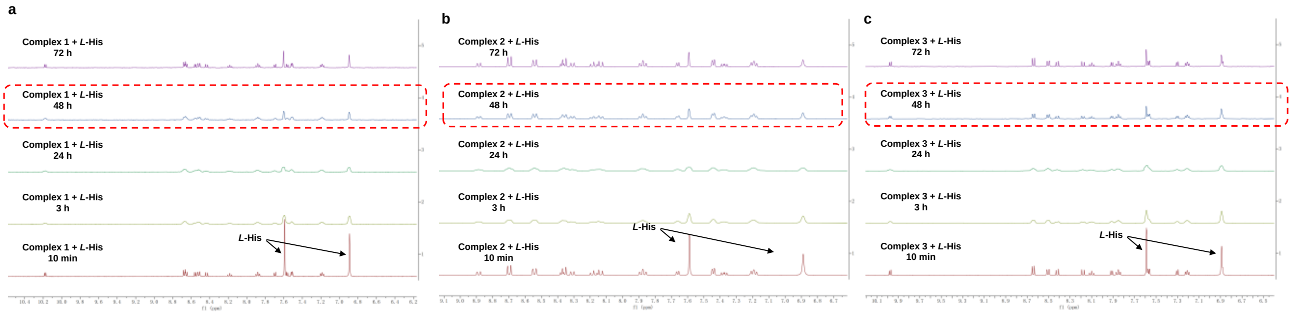

Figure S16. Time-depended ^{1}H NMR spectrum (aromatic part) between complexes and *L*-His (ratio of 1:3) of a) complex **1**, b) complex **2**, and c) complex **3**.

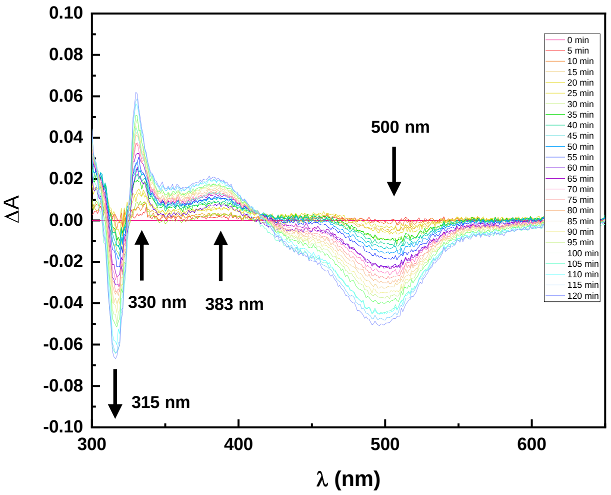

Figure S17. Time evolution of UV-Vis difference spectra during the interaction of the complex **2**.

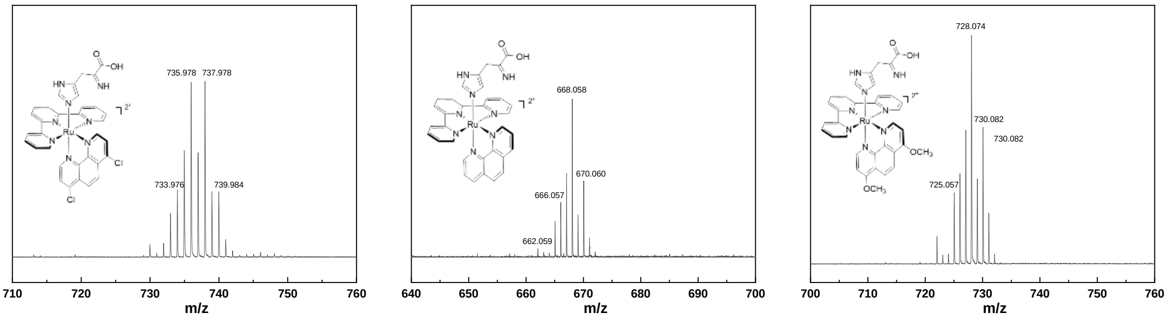

Figure S18. MALDI-TOF mass spectra of adducts **1 - 3**.

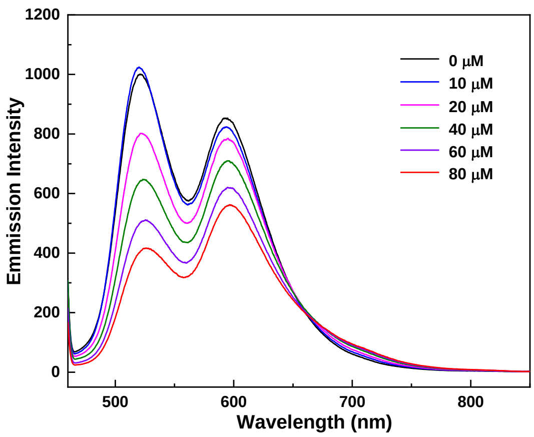

Figure S19. Emission spectra of EB bound to DNA in the presence of complex **2**.

Table S1. The IC_{50} values of complex 1- 3 and cisplatin to six tumor cells.

**Compounds**	**IC_{50} (μM)**					
	**Jurkat**	**U937**	**CCRF-CEM**	**HL60**	**HepG2**	**SMCC7721**
**complex 1**	19.68±4.85	37.00±5.40	22.93±2.09	6.23±0.26	>50	>50
**complex 2**	15.47±9.46	27.72±9.98	14.51±2.99	42.15±2.22	>50	>50
**complex 3**	20.98±1.37	>50	28.34±6.05	>50	>50	>50
**cisplatin**	46.01±2.52	>50	>50	45.53±1.25	>50	>50

Coordinates of the optimized geometries:

complex **1**

Ru	0.79514897	-0.54981917	0.00059394
Cl	1.81752834	-2.75672909	0.00295598
N	1.17839399	-0.42746595	-2.07568055
N	2.62059143	0.24484971	-0.00024620
N	1.17836010	-0.42297934	2.07661126
N	-0.39142185	1.19785749	-0.00130258
N	-1.18324676	-1.34434639	0.00144392
C	0.39079702	-0.82023817	-3.08906614
C	0.77697071	-0.73176991	-4.42352226
C	2.03659214	-0.21588381	-4.72305720
C	2.86453755	0.18651228	-3.67817755
C	2.42202780	0.06877765	-2.35867544
C	3.24430895	0.43735618	-1.18756122
C	4.55622237	0.91716239	-1.21016531
C	5.20621542	1.16697243	-0.00122045
C	4.55618589	0.91981994	1.20825089
C	3.24427295	0.43996483	1.18666120
C	2.42196631	0.07393198	2.35855611
C	2.86443624	0.19455482	3.67781054
C	2.03648724	-0.20562197	4.72354000
C	0.77690831	-0.72224211	4.42509445
C	0.39076960	-0.81362050	3.09082392
C	0.02960891	2.46537660	-0.00268643
C	-0.84110110	3.56184069	-0.00387338
C	-2.20722462	3.34069655	-0.00362237
C	-2.70611683	2.01239544	-0.00217992

C	-1.74276118	0.97177041	-0.00105503
C	-4.09632243	1.66325306	-0.00180082
C	-4.50075467	0.36019610	-0.00039676
C	-3.55148312	-0.71317382	0.00076079
C	-2.16976389	-0.39846210	0.00042438
C	-3.88396856	-2.09337902	0.00224803
C	-2.87892367	-3.04468009	0.00327781
C	-1.53825500	-2.63248783	0.00283477
H	-0.57444646	-1.22466952	-2.80687001
H	0.10174815	-1.06709171	-5.20327200
H	2.37372499	-0.13282046	-5.75131732
H	3.85183141	0.58149931	-3.88844554
H	5.07259453	1.07997015	-2.14890555
H	6.22644075	1.53628790	-0.00161094
H	5.07252958	1.08468606	2.14664736
H	3.85170446	0.59004821	3.88724587
H	2.37359022	-0.12030828	5.75162567
H	0.10169326	-1.05592588	5.20555307
H	-0.57443357	-1.21873790	2.80947387
H	1.10315179	2.61757079	-0.00286268
H	-0.44192311	4.56940038	-0.00497212
H	-4.83136493	2.45951106	-0.00265826
H	-5.55759156	0.12002395	-0.00013802
H	-3.11618668	-4.10227483	0.00441945
H	-0.71734341	-3.34357065	0.00360317
Cl	-5.54538852	-2.60255236	0.00279065
Cl	-3.29159902	4.69745226	-0.00508582

complex **2**

Ru	-0.26782128	-0.00000229	-0.43965414
Cl	-1.21392294	-0.00001602	-2.68214583
N	-0.65371079	-2.07505507	-0.32800491
N	-2.11700531	0.00000330	0.29423393
N	-0.65370775	2.07505053	-0.32803099
N	0.85165971	0.00001007	1.35594027
N	1.73953736	-0.00001025	-1.17217569
C	0.14667071	-3.08816140	-0.69473830
C	-0.24163860	-4.42268483	-0.61789643
C	-1.51712361	-4.72262546	-0.14261235
C	-2.35816105	-3.67799972	0.23210196
C	-1.91268356	-2.35830177	0.12783841
C	-2.74670907	-1.18716370	0.46841115
C	-4.07320159	-1.20917514	0.90620267
C	-4.73090343	0.00001126	1.13384089
C	-4.07319916	1.20919358	0.90618750
C	-2.74670653	1.18717375	0.46839742
C	-1.91267714	2.35830590	0.12781347
C	-2.35814875	3.67800696	0.23206962
C	-1.51710689	4.72262583	-0.14265151
C	-0.24162380	4.42267616	-0.61793681
C	0.14667856	3.08815098	-0.69477305
C	0.39138264	0.00002190	2.61034238
C	1.23651742	0.00003225	3.73156628
C	2.60772538	0.00002922	3.55309713
C	3.13139364	0.00001648	2.24180691
C	2.20860437	0.00000735	1.16745645

C	4.53717298	0.00001073	1.94613578
C	4.98839043	-0.00000312	0.65865570
C	4.07378502	-0.00001150	-0.44863502
C	2.68363178	-0.00000446	-0.18337980
C	4.47527481	-0.00002364	-1.80331805
C	3.51055399	-0.00002866	-2.79409210
C	2.14801005	-0.00002157	-2.44477831
H	1.12394229	-2.80485391	-1.06833120
H	0.44424459	-5.20237479	-0.93104973
H	-1.85628716	-5.75102669	-0.06992576
H	-3.35771685	-3.88838182	0.59495446
H	-4.59438352	-2.14783283	1.05365794
H	-5.76238236	0.00001436	1.47046755
H	-4.59437972	2.14785422	1.05362939
H	-3.35770276	3.88839528	0.59492318
H	-1.85626392	5.75102960	-0.06996957
H	0.44426179	5.20236166	-0.93109643
H	1.12394623	2.80483521	-1.06836892
H	-0.68701630	0.00002294	2.72537906
H	0.79920165	0.00004153	4.72432109
H	5.23919239	0.00001753	2.77487483
H	6.05436072	-0.00000767	0.45005302
H	3.78489309	-0.00003758	-3.84367627
H	1.35528530	-0.00002548	-3.18685841
H	3.28211248	0.00003641	4.40453982
H	5.53265429	-0.00002919	-2.05267294

complex **3**

Ru	0.75367682	-0.58603439	0.00004705
Cl	1.65201411	-2.85197439	0.00024440
N	1.13533728	-0.47641268	-2.07318976
N	2.61193303	0.11123682	0.00001212
N	1.13527414	-0.47604683	2.07327462
N	-0.34785227	1.23031052	-0.00011394
N	-1.27555326	-1.28033410	0.00008713
C	0.32506554	-0.82100379	-3.08646474
C	0.71226886	-0.74811486	-4.42135315
C	1.99792852	-0.30102211	-4.72257220
C	2.84898742	0.05106789	-3.67847924
C	2.40379614	-0.04735678	-2.35795965
C	3.24581772	0.27388235	-1.18761342
C	4.58063510	0.68526452	-1.20914781
C	5.24310737	0.89969879	-0.00001710
C	4.58059639	0.68548311	1.20913147
C	3.24577938	0.27409870	1.18762866
C	2.40372065	-0.04692846	2.35800660
C	2.84886630	0.05174872	3.67852278
C	1.99777453	-0.30015006	4.72265322
C	0.71212889	-0.74731099	4.42147485
C	0.32497164	-0.82045584	3.08658723
C	0.12159217	2.47742265	-0.00021507
C	-0.69254857	3.61821089	-0.00030877
C	-2.07530986	3.46809865	-0.00029861
C	-2.61959975	2.14679589	-0.00019353
C	-1.71104483	1.06682917	-0.00010438

C	-4.02550683	1.88284294	-0.00017688
C	-4.49824701	0.59978694	-0.00007573
C	-3.59917075	-0.51209933	0.00001669
C	-2.20795171	-0.27773866	0.00000218
C	-4.03460591	-1.87405773	0.00012610
C	-3.07458595	-2.88067866	0.00021237
C	-1.71355967	-2.54027484	0.00018939
H	-0.65968267	-1.17291917	-2.80113954
H	0.01788578	-1.04256150	-5.20089345
H	2.33666239	-0.23277085	-5.75146078
H	3.85618351	0.39206827	-3.88936331
H	5.10449469	0.82242183	-2.14798094
H	6.28120070	1.21537725	-0.00002886
H	5.10442679	0.82280842	2.14795642
H	3.85605226	0.39279777	3.88937654
H	2.33647216	-0.23170048	5.75154058
H	0.01772300	-1.04161858	5.20104718
H	-0.65976256	-1.17243644	2.80129457
H	1.20131790	2.58338300	-0.00022404
H	-0.22300302	4.59363911	-0.00038785
H	-4.70891190	2.72369720	-0.00024670
H	-5.56406260	0.40373315	-0.00006378
H	-3.34610486	-3.92875300	0.00029992
H	-0.93814451	-3.30080160	0.00025538
C	-2.49609242	5.82186088	-0.00048648
H	-1.90326415	6.02832578	-0.89841216
H	-3.39168785	6.44251144	-0.00053477
H	-1.90326338	6.02846575	0.89740692

C	-5.87446131	-3.40085911	0.00024520
H	-6.95922858	-3.29776458	0.00023187
H	-5.55385842	-3.94100134	-0.89724383
H	-5.55386631	-3.94085314	0.89782637
O	-5.36238723	-2.06361264	0.00013706
O	-2.96632313	4.47005113	-0.00038108
